# Characteristics of the gut microbiota in women with premenstrual symptoms: A cross-sectional study

**DOI:** 10.1371/journal.pone.0268466

**Published:** 2022-05-27

**Authors:** Takashi Takeda, Kana Yoshimi, Sayaka Kai, Genki Ozawa, Keiko Yamada, Keizo Hiramatsu

**Affiliations:** 1 Division of Women’s Health, Research Institute of Traditional Asian Medicine, Kindai University, Osaka-Sayama, Osaka, Japan; 2 TechnoSuruga Laboratory Co. Ltd., Shizuoka, Japan; 3 Department of Anesthesiology and Pain Medicine, Juntendo University Faculty of Medicine, Tokyo, Japan; 4 Hiramatsu Women’s Clinic, Fujiidera, Osaka, Japan; University of California Los Angeles, UNITED STATES

## Abstract

**Purpose:**

Premenstrual symptoms can negatively impact the quality of life of women through a range of mood, behavioral, and physical symptoms. The association between the microbiota and brain function has been extensively studied. Here, we examined the characteristics of the microbiota in women with premenstrual disorders (PMDs) and the association between premenstrual symptoms and the microbiota.

**Materials and methods:**

In this single center cross-sectional pilot study, we recruited 27 women reporting premenstrual symptoms and 29 women with no serious premenstrual symptoms. Among them, we further selected 21 women experiencing premenstrual symptoms resulting in interference to their social life (PMDs group) and 22 women with no serious premenstrual symptoms and thereby no interference to their social life (control group). The severity of symptoms was evaluated by a premenstrual symptoms questionnaire (PSQ). Inflammatory markers were analyzed in blood samples, including C reactive protein, soluble CD14, and lipopolysaccharide binding protein. Sequencing of 16S ribosomal ribonucleic acid genes was performed on stool samples.

**Results:**

Inflammatory markers in blood samples did not differ significantly between the PMDs and control groups. A difference in beta, but not alpha diversity, was detected for the gut microbiotas of the PMDs and control groups. The relative abundance of the *Bacteroidetes* phylum was lower in the PMDs group. At the genus level, the prevalence was decreased for *Butyricicoccus*, *Extibacter*, *Megasphaera*, and *Parabacteroides* and increased for *Anaerotaenia* in the PMDs group, but after false discovery rate correction, these differences were no longer significant. Linear discriminant effect size analysis revealed a decrease in *Extibacter*, *Butyricicoccus*, *Megasphaera*, and *Parabacteroides* and an increase in *Anaerotaenia* in the PMDs group. The PSQ total score correlated with *Anaerotaenia*, *Extibacter*, and *Parabacteroides*. Multiple regression analysis showed that *Parabacteroides* and *Megasphaera* negatively predicted the PSQ total score.

**Conclusion:**

The properties of the gut microbiota are associated with premenstrual symptoms.

## Introduction

Premenstrual symptoms encompass a range of psycho-physical symptoms observed before menstruation, which interfere with the quality of life of many women between menarche and menopause [1–3]. In epidemiologic surveys, the prevalence of premenstrual symptoms is high (80%–90%) [4]. As a disease, it has been classified as premenstrual syndrome (PMS) by the field of obstetrics and gynecology and as premenstrual dysphoric disorder (PMDD) by the field of psychiatry, but recently both have been recognized by the inclusive term, premenstrual disorders (PMDs) [5]. Various causes have been suggested, including hormonal changes, serotonergic dysfunction, impaired gamma-aminobutyric acid (GABA) function, stress, and poor lifestyle habits such as longer durations of internet use and shorter sleep durations, but the precise pathophysiology of PMDs remains unknown [6–9].

As “another organ”, the gut microbiota exhibits complex interactions with the immune, metabolic, and endocrine systems through the host’s intestinal epithelium, and maintains a delicate balance [10–12]. Many important diseases such as obesity, metabolic disease, cardiovascular disease, inflammatory diseases, and brain disorders are found to be associated with differences in the gut microbiome [13, 14]. Molecular methods, especially amplicon sequence analysis using next generation sequencing, have enabled us to detect the diversity and composition of the gut microbiota in detail.

Recently, the association between the microbiota and brain function, such as in the case of major depressive disorder (MDD), has been extensively studied [15–18]. The gut microbiota communicates with the brain through neuroendocrine, neuroimmune, and neural pathways, and this is known as the gut microbiota–brain axis [14, 19–21]. According to clinical data, MDD is associated with low-grade inflammation (C reactive protein (CRP) >3 mg/L) [22, 23]. One possible mechanism may be microbiota-related inflammation, or so-called “leaky gut” [24]. A dysfunctional intestinal barrier may permit the translocation of Gram-negative bacteria and the bacterial endotoxin (lipopolysaccharide, LPS) from the gut microbiota to the bloodstream [24]. Bacterial translocation induces LPS-binding protein (LBP) and soluble CD14 (sCD14) that potentiates inflammation through Toll-like receptor (TLR)-4 and NF-κB activation [24]. LBP and sCD14 may be markers of endotoxemia and are reported to be associated with depressive symptoms [25, 26].

PMDs are linked to various mood and behavioral symptoms, which overlap with MDD. For treatment, serotonin reuptake inhibitors are recommended and commonly prescribed for both PMDs and MDD [2, 27–29]. Despite the commonality between PMDs and MDD, there have been no reports to date profiling the microbiota in PMDs. Our goal in this pilot study was to examine the possibility that characteristics of the gut microbiota may be related to the pathogenesis of PMDs. The aims of this study were to: (1) test the association between PMDs and bacterial translocation, (2) study the characteristics of the gut microbiota in women with PMDs, and (3) study the association between the gut microbiota and premenstrual symptom severity.

## Materials and methods

### Ethics approval and informed consent

The study was carried out in accordance with the principles outlined in the Declaration of Helsinki. The trial protocol was approved by the Ethics Committee of Kindai University (approval number: 31–057). Written informed consent was obtained from all participants.

### Settings and participants

**Fig 1:**
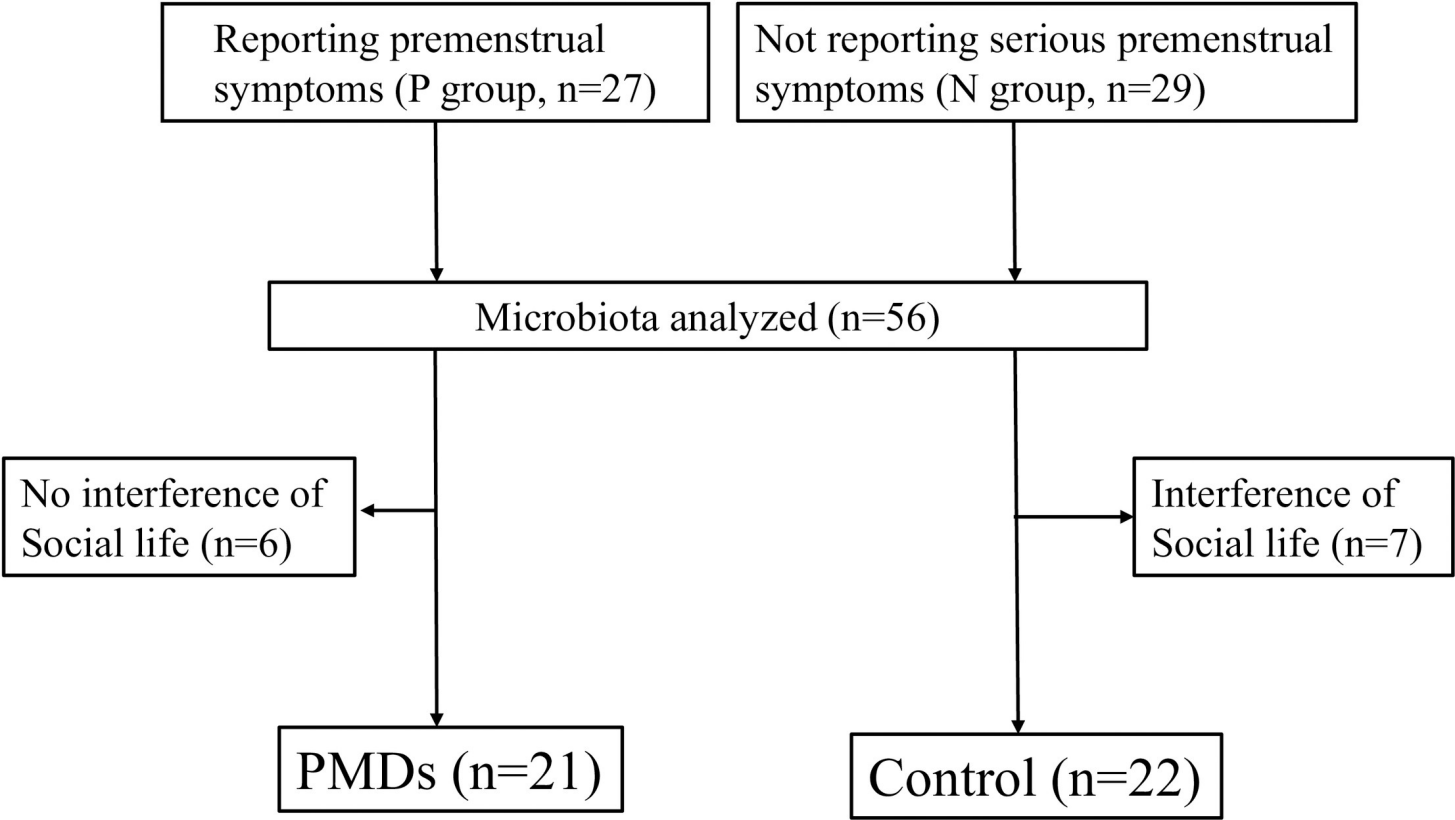
Flow chart of the study design. **Abbreviation:** PMDs, premenstrual disorders.

This study was conducted at an obstetrics and gynecology outpatient clinic in Osaka, Japan. Study participants were enrolled between September 2019 and August 2020. We recruited patients who wished to receive treatment for their premenstrual symptoms (P group, n = 27) and healthy volunteers who did not report serious premenstrual symptoms (N group, n = 29) (Fig 1). The inclusion criteria were: aged from 20 to 45 years, not receiving treatment for premenstrual symptoms, regular menstrual cycles (25 to 38 days), no neuropsychiatric disorders, and had not taken drospirenone-containing oral contraceptive (OC)s, antidepressants, herbal medicine, probiotics, or antibiotics for 4 weeks before study entry. Drospirenone-containing OCs were excluded because, unlike other conventional OCs, they have a proven therapeutic effect on PMDs [30]. In addition, the inclusion criteria for the P group included at least one or more of the symptoms listed in the premenstrual symptoms questionnaire (PSQ) showing a moderate or higher level, and for the N group included no symptoms listed in the PSQ showing a moderate or higher level. In total, the microbiotas of 56 patients were analyzed. None of these patients had a history of inflammatory bowel disease, irritable bowel disease, malignancy, any gastrointestinal tract surgery, or diabetes, and all were OC non-users. According to the criteria for PMDs defined by the International Society of Premenstrual Disorder, there is no regulation on the number of premenstrual symptoms, but marked interference to the patient’s social life by the premenstrual symptoms is essential [5]. For an accurate diagnosis of PMD, it is recommended to keep a symptoms diary for two prospective periods, but this is not common in general gynecological practice in Japan. In this study, we further selected suspected cases of PMD (PMDs group) (n = 21) from the P group as those who experienced interference to their social life by the premenstrual symptoms. We also selected a control group (n = 22) from the N group who did not experience interference to their social life by premenstrual symptoms. The seven patients excluded from the N group had multiple mild premenstrual symptoms and mild interference to their social life due to these symptoms, as assessed by the PSQ.

### Questionnaire

#### The premenstrual symptoms questionnaire

In this study, we used the PSQ, developed in our previous study [31], to evaluate the severity of premenstrual symptoms. The PSQ has been found to have high reliability and validity [32].

The PSQ asks, “Within the last 3 months, have you experienced the following premenstrual symptoms starting during the week before menses and stopping a few days after the onset of menses?” The premenstrual symptoms listed are as follows: (i) depressed mood, (ii) anxiety or tension, (iii) tearfulness, (iv) anger or irritability, (v) decreased interest in work, home, or social activities, (vi) difficulty concentrating, (vii) fatigue or lack of energy, (viii) overeating or food cravings, (ix) insomnia or hypersomnia, (x) feeling overwhelmed, and (xi) physical symptoms such as tender breasts, feeling of bloating, headache, joint or muscle pain, or weight gain. These 11 symptoms are listed in the diagnostic criteria for PMDD in the *Diagnostic and Statistical Manual of Mental Disorders* (DSM). Furthermore, the PSQ asks whether these symptoms interfere with (a) work efficiency or productivity, or home responsibilities; (b) social activities; or (c) relationships with coworkers or family. The participants were asked to rate the severity of premenstrual symptoms and the interference with daily activities resulting from these symptoms as *1—Not at all*, *2—Mild*, *3—Moderate*, or *4—Severe*. The total scores of the PSQ were calculated as the sum of the 14 items. The PSQ total score was applied for the evaluation of premenstrual symptoms severity and ranges from 14 to 56.

#### Menstrual pain intensity

A numerical rating scale (NRS) was applied for the evaluation of menstrual abdominal pain intensity. On the NRS, study participants rate their severity of menstrual pain from 0 (*no pain*) to 10 (*maximum pain you can imagine*).

#### Evaluation of basic attributes

For each participant, we also collected information about age, parity, body weight, height, age at menarche, total sleep duration, smoking (yes or no), drinking (yes or no), regular exercise (yes or no), diarrhea or constipation. Body mass index (kg/m^2^) was calculated by dividing weight in kilograms by height in meters squared.

### Measurement of inflammatory markers in blood samples

Blood samples were collected immediately after the PSQ and NRS assessment. Serum-separated samples were stored at −20°C until further analysis. Frozen samples were transferred to Filgen, Inc. (Nagoya, Japan) and CRP, soluble CD14 (sCD14), and lipopolysaccharide binding protein (LBP) were analyzed using the Human Magnetic Luminex Assay (R&D Systems, Inc., Minneapolis, MN, USA) according to the manufacturer’s protocols. All specimens were measured in triplicate and the average was obtained.

### Analysis of the microbiota

All subjects were instructed to collect a stool sample at home. The participants scraped the surface of their stool with a swab after defecation and collected stool specimens in sampling tubes containing guanidine thiocyanate solution (Techno Suruga Laboratory Co. Ltd., Shizuoka, Japan). The DNA preservation solution ensures that the DNA of the stool specimen in the sampling tube is stable for at least one month at room temperature or at 4°C. The specimens were shipped by the subjects to our laboratory the same day using an express courier service, arriving no later than 48 hours after specimen collection. The collected specimens were sent to Techno Suruga Laboratory using a courier service at 4°C. The samples were stored at 4°C before DNA extraction. Extracted bacterial DNA was subjected to amplicon sequence analysis using the MiSeq system (Illumina, San Diego, CA, USA) by Techno Suruga Laboratory, as described previously [33]. Then, DNA was extracted using an automated DNA isolation system (GENE PREP STAR PI-480, Kurabo, Osaka, Japan), with 200 μL of distilled water being included as a negative control sample. The V3-V4 regions of Bacterial and Archaea 16S rRNA were amplified from the extracted DNA using the Pro341F/Pro805R primers and the dual-index method [33, 34], a negative control sample was also included, and the amplicons were visualized by electrophoresis. Barcoded amplicons were paired-end sequenced using a 2×284-bp cycle and the MiSeq system with MiSeq Reagent Kit version 3 (600 Cycle) chemistry. The quality of the paired-end sequencing reads was checked using the FASTX-Toolkit [35]. Paired-end sequencing reads were merged using the fastq-join program with default settings [36]. Only joined-reads that had a quality value score (QC) of ≥ 20 for more than 99% of the sequence were extracted using the FASTX-Toolkit [35]. Chimeric sequences were deleted with usearch6.1 [37, 38]. Bacterial and Archaea species identification from sequences was performed using the Metagenome@KIN Ver 2.2.1 analysis software (World Fusion, Japan) and the TechnoSuruga Lab Microbial Identification database DB-BA 13.0 (TechnoSuruga Laboratory, Japan) with homology of ≥ 97% [39]. The relative abundance of each bacterium at the phylum and genus level was calculated.

The 16S rRNA data were also analyzed with Quantitative Insights into Microbial Ecology (QIIME) 2.0 ver. 2020.6 [40]. Quality filtering and chimeric sequences were filtered using DADA2 (Divisive Amplicon Denoising Algorithm 2) denoise-single plugin ver. 2017.6.0 with default option [41]. Taxonomy was assigned using Greengenes database ver. 13.8 based on an average percent identity of 99% [42] by training a Naive Bayes classifier using the q2-feature-classifier plugin. To analyze beta diversity, weighted unifrac distance metrics were used. We used principal coordinates analysis (PCoA) to show the pattern of differences in the PMDs group and the control group. Alpha diversity was calculated by the Chao 1 [43], Shannon [44], and Simpson indices [45]. To further investigate the differences in abundance between the PMDs group and control group at the genus level, linear discriminant effect size analysis (LEfSe) was performed through the Huttenhower Lab Galaxy Server [46]. LEfSe was performed under the following conditions: the alfa value for the Kruskal–Wallis test was 0.05 and the threshold for the logarithmic linear discriminant analysis (LDA) score for a discriminative feature was 2.0.

### Statistical analysis

For continuous variables, normally distributed data were expressed as means and standard deviations and were analyzed by the Student’s *t*-test, while non-normally distributed data were expressed as medians and interquartile ranges and were analyzed by the Wilcoxon signed-rank test. For the Student’s *t*-test, effect size was measured using *r*, and *r* was calculated by the following formula (*r* = $\surd \frac{t2}{t2+df}$). For the Wilcoxon signed-rank test, effect size was measured using *r*, and *r* was calculated by the following formula (*r* = Z/√N). For categorical variables, proportions were calculated and analyzed by Fisher’s exact test. Effect size was measured using Cramer’s V. The effect sizes of 0.10, 0.30, and 0.50 were judged as small, medium, and large, respectively [47].

For the relative abundance analysis of gut microbiota, multiple comparisons were adjusted using false discovery rate (FDR) correction. An FDR-adjusted *P* value (*q* value) < 0.20 was set as the cut-off [48].

Correlations between gut microbiota abundance and PSQ total score were analyzed using Spearman’s rank correlation coefficient. Multiple regression analysis was used to explore the association between the microbiota and the PSQ total score. Variables that were predictive at a *P* value less than 0.20 were introduced into the stepwise model.

Statistical analyses were performed using JMP Pro 15.1.0 (SAS, Cary, NC, USA), except for the relative abundance analysis of the gut microbiota, for which SAS 9.4 (SAS) was used. Statistical significance was set at *P* < 0.05 (for two-tailed tests).

## Results

The characteristics of the study population are presented in Table 1.

**Table 1:** Characteristics of the study participants.

Characteristic	Total (n = 56)	PMDs (n = 21)	Control (n = 22)	*P*
Age (years), median (IQR)	27.5 (23.0–35.0)	26.0 (23.0–31.0)	26.0 (22.8–37.5)	0.394^a^ (*r* = 0.130)
Parity, median (IQR)	0 (0–1.8)	0 (0–2.0)	0 (0–0)	0.073^a^ (*r* = 0.273)
Age at menarche (years), median (IQR)	12.0 (11.0–13.0)	12.0 (11.0–13.0)	12.5 (11.0–14.0)	0.397^a^ (*r* = 0.129)
BMI (kg/m^2^), median (IQR)	21.7 (19.3–23.1)	21.2 (19.1–22.7)	20.4 (19.0–23.4)	0.799^a^ (*r* = 0.039)
Menstrual pain intensity, median (IQR)	4.5 (3.0–7.0)	8.0 (5.5–8.0)	3.0 (1.0–4.0)	<0.0001^a^ (*r* = 0.656)
Total sleep duration (hours), median (IQR)	6.0 (6.0–7.0)	6.0 (6.0–7.0)	6.3 (6.0–7.0)	0.207^a^ (*r* = 0.192)
Smoker, n (%)	9.0 (16.1)	1.0 (4.8)	6.0 (27.3)	0.095^b^ (*V* = 0.305)
Drinker, n (%)	19.0 (33.9)	6.0 (28.6)	9.0 (40.9)	0.526^b^ (*V* = 0.129)
Regular exercise, n (%)	7.0 (12.7)	3.0 (15.0)	1.0 (4.6)	0.333^b^ (*V* = 0.178)
Diarrhea or constipation, n (%)	23.0 (41.1)	10.0 (47.6)	7.0 (31.8)	0.358^b^ (*V* = 0.162)
PSQ total score, median (IQR)	22.0 (18.0–30.0)	32.0 (28.5–40.5)	17.0 (15.8–18.3)	<0.0001^a^(*r* = 0.844)

**Abbreviations:** PMDs, premenstrual disorders; IQR, interquartile range; BMI, body mass index; SD, standard deviation; PSQ, premenstrual symptoms questionnaire; V, Cramer’s V

^a^ Wilcoxon signed-rank test

^b^ Fisher’s exact test

The severity of menstrual pain was stronger and the PSQ total score was higher in the PMDs group than in the control group (*P* < 0.0001).

The differences in endotoxin biomarkers between the two groups are presented in Table 2.

**Table 2:** Comparison of the endotoxin biomarkers present in the PMDs group and the control group.

Characteristic	PMDs (n = 21)	Control (n = 22)	*P*
CRP (ng/ml), median (IQR)	308.2 (117.0–477.6)	242.3 (61.1–980.6)	0.743^a^ (*r* = 0.050)
s-CD14 (ng/ml), mean (SD)	918.2 (37.7)	912.7 (36.8)	0.917^b^ (*r* = 0.016)
LBP (ng/ml), median (IQR)	5859.6 (5213.7–6579.0)	5404.2 (4506.6–7312.7)	0.536^a^ (*r* = 0.093)

**Abbreviations:** PMDs, premenstrual disorders; CRP, C reactive protein; IQR, interquartile range; s-CD14, soluble cluster of differentiation 14; SD, standard deviation; LBP, lipopolysaccharide binding protein

^a^ Wilcoxon signed-rank test

^b^ Student’s *t*-test

There was no significant difference between the expression levels of endotoxin biomarkers in the PMDs group and the control group.

**Fig 2:**
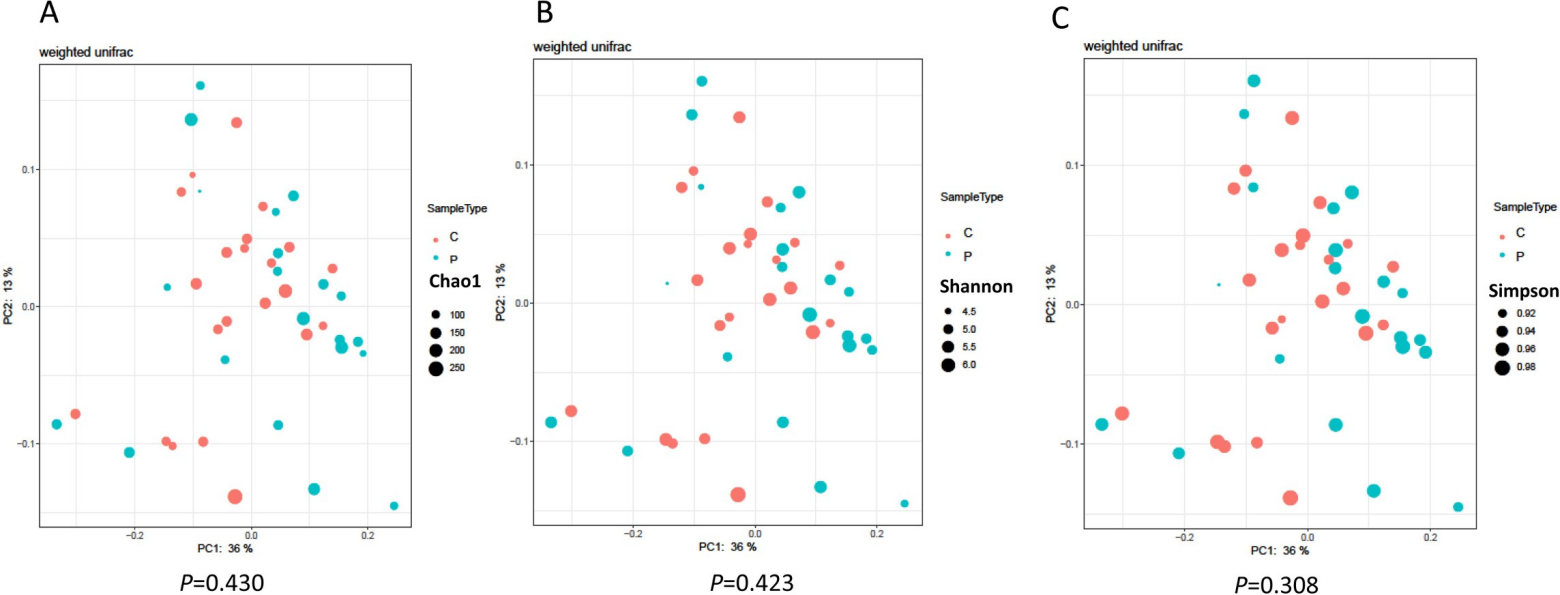
Principal coordinates analysis plot comparing sample distribution between the PMDs group and the control group. Each blot shows data for alpha diversity, the Chao 1 index (A), the Shannon index (B), and the Simpson index (C). **Abbreviations:** C, control group; P, premenstrual disorders group.

Next, we analyzed alpha and beta diversity in the two groups (Fig 2). Regarding alpha diversity, there were no significant differences between the PMDs group and the control group as determined by the Chao 1, Shannon, and Simpson indices (*P* = 0.430, 0.423, and 0.308, respectively). Regarding beta diversity, as analyzed by PCoA, a significant difference was detected between the PMDs group and the control group (*R* = 0.062, *P* = 0.027).

**Fig 3:**
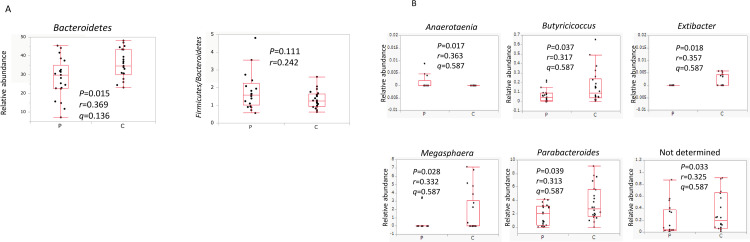
Comparison of the gut microbiotas between the PMDs group and the control group. The relative abundance of each taxon in the gut microbiota was compared. (A) At the phylum level, only Bacteroidetes was significantly less abundant in the PMDs group than in the control group. (B) The abundance of genus-level bacteria was significantly different between the PMDs group and the control group. The Wilcoxon signed-rank test was used to compare differences between the two groups (*P* < 0.05). **Abbreviations:** PMDs, premenstrual disorders; P, premenstrual disorders group; C, control group.

The relative abundance of organisms in the gut microbiota was compared between the PMDs group and the control group (Fig 3). At the phylum level, the PMDs group possessed fewer *Bacteroidetes* than the control group (*P* = 0.015, *q* = 0.136) (Fig 3A). We further analyzed the *Firmicutes/Bacteroidetes* (F/B) ratio, but this did not differ significantly between the two groups (*P* = 0.111). At the genus level, the PMDs group had a lower prevalence of *Butyricicoccus*, *Extibacter*, *Megasphaera*, *Parabacteroides*, and “Not determined” (*P* = 0.037, 0.018, 0.028, 0.039 and 0.033, respectively), and a higher prevalence of *Anaerotaenia* (*P* = 0.017) than the control group; however, after FDR correction, this significance was lost (Fig 3B).

**Fig 4:**
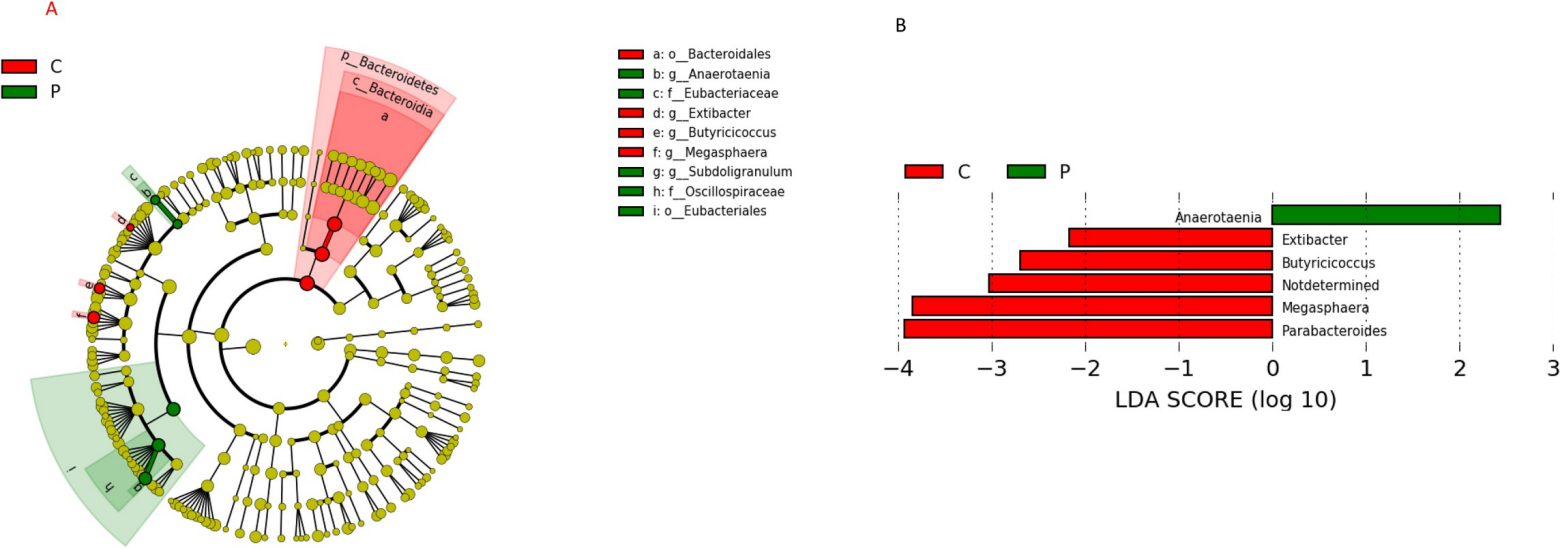
Linear discriminant effect size analysis to distinguish the differential microbiota between the PMDs group and control group. (A) Cladogram showing the most differentially abundant taxa between the PMDs group (P) and control group (C). Taxa enriched in the PMDs group are indicated in green and taxa enriched in the control group are indicated in red. The brightness of each dot is proportional to the respective effect size. (B) Comparison of the LDA scores between the P and C groups at the genus level. **Abbreviations:** LDA, logarithmic linear discriminant analysis; PMDs, premenstrual disorders; P, premenstrual disorders group; C, control group.

Furthermore, we analyzed the characteristic gut microbiota differences in the PMDs group and the control group by LEfSe (Fig 4A and 4B). At the genus level, *Anaerotaenia* was enriched in the PMDs group, whereas *Extibacter*, *Butyricicoccus*, “Not determined”, *Megasphaera*, and *Parabacteroides* were enriched in the control group (Fig 4B).

Next, we analyzed the association between the abundance of organisms in the gut microbiota and the severity of premenstrual symptoms (Table 3).

**Table 3:** Correlation analysis between the gut microbiota abundance and the PSQ total score (n = 56).

	*R*	*P*
*Anaerotaenia*	0.292	0.029
*Extibacter*	−0.410	0.002
*Parabacteroides*	−0.342	0.010

**Abbreviations:** PSQ, premenstrual symptoms questionnaire

At the genus level, the PSQ total score was positively associated with *Anaerotaenia*, and negatively associated with *Extibacter* and *Parabacteroides*. Multiple regression analysis showed that the PSQ total score was negatively associated with *Parabacteroides* and *Megasphaera* (Table 4).

**Table 4:** Multiple regression analysis calculating the associations between the microbiota and the PSQ total score (n = 56).

	β	95% CI	*P*	Standardized β	VIF
*Blautia*	0.36	−0.07 to 0.79	0.10	0.23	1.24
*Faecalibacterium*	−0.33	−0.82 to 0.17	0.19	−0.17	1.07
*Parabacteroides*	−1.31	−2.43 to -0.18	0.02	−0.30	1.14
*Ruminococcus*	1.15	−0.07 to 2.36	0.06	0.25	1.27
*g_Lachnospiraceae bacterium KNHs209_incertae_sedis*	−2.65	−5.36 to 0.06	0.06	−0.24	1.10
*Megasphaera*	−1.56	−2.89 to -0.24	0.02	−0.31	1.19

*R*^*2*^ = 0.29

**Abbreviations:** PSQ, premenstrual symptoms questionnaire; β, regression coefficient; CI, confidence interval; VIF, variance inflation factor

Variance inflation factor analysis showed that multicollinearity was not present for premenstrual symptoms in this model.

## Discussion

To our knowledge, this is the first report to investigate the association between the gut microbiota and premenstrual symptoms. By amplicon sequencing, we detected a difference in the gut microbiota between the PMDs group and the control group. Furthermore, we identified several organisms within the gut microbiota that were significantly associated with the severity of premenstrual symptoms.

In the case of MDD, increased bacterial translocation has been suggested to play a role in the inflammatory pathophysiology [24]. In this study, rather than directly assessing bacterial translocation by analyzing the presence of gut bacteria in the blood, we instead indirectly assessed bacterial translocation by measuring the levels of inflammatory factors CRP, LBP, and sCD14. LBP and sCD14 are produced in response to bacterial translocation and are proposed markers of endotoxemia [24]. Given the exploratory nature of this study, we chose the indirect method of evaluation as a simpler method for this initial investigation. According to our data, these indicators of inflammation did not show any correlation with PMDs. MDD and PMDs are closely related in terms of their clinical symptoms. However, the duration of symptoms is distinct between these two disorders, with MDD symptoms being permanent and PMD symptoms being temporary. This may explain the difference in the degree of inflammation between MDD and PMDs.

There was no significant difference in alpha diversity between the PMDs group and the control group. According to data from meta-analysis of MDD patients, there was no significant difference in alpha diversity between MDD patients and controls [18]. In other diseases, such as inflammatory bowel disease, obesity, and metabolic diseases, decreased microbiota diversity was suggested to be associated with the development of these diseases [49, 50]. This may explain some of the common pathology of MDD and PMDs.

At the phylum level, *Bacteroidetes* was less abundant in the PMDs group than in the control group. Low *Bacteroidetes* levels have been reported to be associated with obesity and MDD [51, 52]. Furthermore, obesity was reported to be a risk factor for PMDs and MDD [53, 54]. Considering that, in this study, no significant difference was found in BMI between the PMDs group and control group, obesity did not seem to be a confounding factor.

At the genus level, our data indicated that, in general, decreased levels of *Butyricicoccus*, *Extibacter*, *Megasphaera*, and *Parabacteroides* were associated with PMDs. These gut microorganisms were different from those reported to be decreased in MDD patients compared with non-MDD patients [18].

Among the gut microbiota, *Butyricicoccus* is a butyrate-producing beneficial bacterium and *Megasphaera* metabolizes lactate to butyrate [55]. In animal models, butyrate-treated mice possessed an increased amount of brain-derived neurotrophic factor (BDNF), which is essential for nerve cell growth and has been linked to antidepressant effects [56]. Decreased levels of butyrate-producing bacteria, such as *Butyricicoccus* and *Megasphaera*, may be involved in the pathology of PMDs.

Decreased levels of *Butyricicoccus* have been reported in postpartum depressive disorder (PPD) [57]. PPD is a unique subtype of MDD, for which the precise pathogenesis remains unknown. During pregnancy, women possess high levels of estrogen and progesterone because of the presence of the placenta and fetus. Dramatic hormonal fluctuations occur after delivery, which is thought to be one of the causes of PPD [58]. The hormonal fluctuations observed with PPD are the same as for PMDs, and premenstrual symptoms have been proposed as a risk factor for PPD [59]. Decreased levels of *Butyricicoccus* may explain some of the common pathology of PPD and PMDs.

*Parabacteroides* is reported to be beneficial in protecting against multiple sclerosis [60], seizures [61], metabolic dysfunctions [62], and colon tumors [63]. Regarding the gut microbiota–brain axis, *Parabacteroides* was reported to be a GABA-producer according to the results of GABA-dependent co-culture assays and *in silico* analyses [64]. In a seizure mouse model, the anti-seizure effects of a ketogenic diet were analyzed in the gut microbiota [61]. In this study, *Parabacteroides* was shown to modulate brain GABA levels. Because impaired GABA function is one of the possible causes of PMDs [7], decreased levels of *Parabacteroides* may be involved in PMD pathology.

Our study had several limitations. The main limitation was that the study was cross-sectional in design. It was therefore impossible to determine causality between premenstrual symptoms and the gut microbiota changes. To clarify the causality, further longitudinal studies are required. Second, our study had a small sample size; however, we believe this was appropriate for a pilot study. Third, we selected the participants from outpatients seeking care. This is a highly select population, which could have led to data bias. Fourth, we selected the PMDs group without prospective daily charting over two consecutive symptomatic cycles, which is recommended by the DSM [30]. However, prospective daily charting is difficult in clinical settings. According to a report from the USA, only 11.5% of physicians reported routinely monitoring two consecutive symptomatic cycles [65]. Fifth, the timing of blood and stool sample collection was performed without considering the menstrual cycle. There is a possibility that the gut microbiota may fluctuate depending on the stage of the menstrual cycle, and sampling at a consistent stage of the menstrual cycle may be necessary in future investigations. Sixth, the study was conducted only in Japan, which might limit the generalization of the findings to the other countries, especially western countries. Finally, the significant difference in the relative abundance of the gut microbiota between the PMDs group and the control group was lost after FDR correction. Considering that the results of LEfSe showed the same pattern of differences in abundance between the PMDs group and the control group, these differences in the gut microbiota between the two groups are likely to be meaningful. Further studies considering these factors are needed to confirm the reliability of these findings. Consecutive prospective evaluations using the Daily Record of Severity of Problem (DRSP) are recommended for accurate assessment of premenstrual symptoms [30], and it is possible to use the Japanese version of the DRSP, for which the validity and reliability have been confirmed [66]. Using DRSP assessment in a large-scale prospective study of the general public, and performing blood and stool collection at specific times during the follicular and luteal phases, respectively, would be expected to provide more definitive results.

Despite these limitations, differences in the gut microbiota between PMD patients and healthy individuals may be applied as biomarkers for diagnosis in the future.

## Conclusions

The present study showed that gut microbial properties were associated with premenstrual symptoms. Decreased levels of *Parabacteroides* and *Megasphaera* are a characteristic feature of PMD patients and are negatively associated with the severity of premenstrual symptoms.

## Supporting information

S1 File Data for Table 1. (XLSX)

S2 File Data for Table 2. (XLSX)

S3 File Phylum-level operational taxonomic units of the fecal microbiota. (XLSX)

S4 File Genus-level operational taxonomic units of the fecal microbiota. (XLSX)

## References

[1] Yonkers KA, O’Brien PM, Eriksson E. Premenstrual syndrome. Lancet. 2008;371(9619):1200–10. Epub 2008/04/09. doi: 10.1016/S0140-6736(08)60527-9 ; PubMed Central PMCID: PMC3118460. 18395582 PMC3118460

[2] Yonkers KA, Simoni MK. Premenstrual disorders. Am J Obstet Gynecol. 2018;218(1):68–74. Epub 2017/06/03. doi: 10.1016/j.ajog.2017.05.045 . 28571724

[3] Takeda T, Koga S, Yaegashi N. Prevalence of premenstrual syndrome and premenstrual dysphoric disorder in Japanese high school students. Arch Womens Ment Health. 2010;13(6):535–7. Epub 2010/09/22. doi: 10.1007/s00737-010-0181-3 . 20857152

[4] Angst J, Sellaro R, Merikangas KR, Endicott J. The epidemiology of perimenstrual psychological symptoms. Acta psychiatrica Scandinavica. 2001;104(2):110–6. Epub 2001/07/28. doi: 10.1034/j.1600-0447.2001.00412.x . 11473504

[5] O’Brien PM, Backstrom T, Brown C, Dennerstein L, Endicott J, Epperson CN, et al. Towards a consensus on diagnostic criteria, measurement and trial design of the premenstrual disorders: the ISPMD Montreal consensus. Archives of women’s mental health. 2011;14(1):13–21. Epub 2011/01/13. doi: 10.1007/s00737-010-0201-3 ; PubMed Central PMCID: PMC4134928. 21225438 PMC4134928

[6] Grady-Weliky TA. Clinical practice. Premenstrual dysphoric disorder. The New England journal of medicine. 2003;348(5):433–8. Epub 2003/01/31. doi: 10.1056/NEJMcp012067 . 12556546

[7] Hantsoo L, Epperson CN. Allopregnanolone in premenstrual dysphoric disorder (PMDD): Evidence for dysregulated sensitivity to GABA-A receptor modulating neuroactive steroids across the menstrual cycle. Neurobiol Stress. 2020;12:100213. Epub 2020/05/22. doi: 10.1016/j.ynstr.2020.100213 ; PubMed Central PMCID: PMC7231988. 32435664 PMC7231988

[8] Yoshimi K, Shiina M, Takeda T. Lifestyle Factors Associated with Premenstrual Syndrome: A Cross-sectional Study of Japanese High School Students. J Pediatr Adolesc Gynecol. 2019;32(6):590–5. Epub 2019/09/14. doi: 10.1016/j.jpag.2019.09.001 . 31518647

[9] Takeda T, Yoshimi K, Imoto Y, Shiina M. Associations between sleep habits and interference of premenstrual symptoms in athletic performance in Japanese adolescent athletes: a cohort study over a 2-year period. Gynecol Endocrinol. 2020;36(10):885–9. doi: 10.1080/09513590.2020.1734787 PubMed PMID: WOS:000518314200001. 32124639

[10] Cox AJ, West NP, Cripps AW. Obesity, inflammation, and the gut microbiota. The Lancet Diabetes & Endocrinology. 2015;3(3):207–15. doi: 10.1016/s2213-8587(14)70134-2 25066177

[11] Dickson RP. The microbiome and critical illness. The Lancet Respiratory Medicine. 2016;4(1):59–72. doi: 10.1016/S2213-2600(15)00427-0 26700442 PMC4752077

[12] Rook G, Bäckhed F, Levin BR, McFall-Ngai MJ, McLean AR. Evolution, human-microbe interactions, and life history plasticity. The Lancet. 2017;390(10093):521–30. doi: 10.1016/s0140-6736(17)30566-4 28792414

[13] Fan Y, Pedersen O. Gut microbiota in human metabolic health and disease. Nat Rev Microbiol. 2021;19(1):55–71. Epub 2020/09/06. doi: 10.1038/s41579-020-0433-9 . 32887946

[14] Morais LH, Schreiber HLt, Mazmanian SK. The gut microbiota-brain axis in behaviour and brain disorders. Nat Rev Microbiol. 2021;19(4):241–55. Epub 2020/10/24. doi: 10.1038/s41579-020-00460-0 . 33093662

[15] Dinan TG, Stanton C, Cryan JF. Psychobiotics: a novel class of psychotropic. Biol Psychiatry. 2013;74(10):720–6. Epub 2013/06/14. doi: 10.1016/j.biopsych.2013.05.001 . 23759244

[16] Aizawa E, Tsuji H, Asahara T, Takahashi T, Teraishi T, Yoshida S, et al. Possible association of Bifidobacterium and Lactobacillus in the gut microbiota of patients with major depressive disorder. J Affect Disord. 2016;202:254–7. Epub 2016/06/12. doi: 10.1016/j.jad.2016.05.038 . 27288567

[17] Tian P, Wang G, Zhao J, Zhang H, Chen W. Bifidobacterium with the role of 5-hydroxytryptophan synthesis regulation alleviates the symptom of depression and related microbiota dysbiosis. J Nutr Biochem. 2019;66:43–51. Epub 2019/02/12. doi: 10.1016/j.jnutbio.2019.01.007 . 30743155

[18] Sanada K, Nakajima S, Kurokawa S, Barcelo-Soler A, Ikuse D, Hirata A, et al. Gut microbiota and major depressive disorder: A systematic review and meta-analysis. J Affect Disord. 2020;266:1–13. Epub 2020/02/15. doi: 10.1016/j.jad.2020.01.102 . 32056863

[19] Cryan JF, Dinan TG. Mind-altering microorganisms: the impact of the gut microbiota on brain and behaviour. Nat Rev Neurosci. 2012;13(10):701–12. Epub 2012/09/13. doi: 10.1038/nrn3346 . 22968153

[20] Bauer KC, Huus KE, Finlay BB. Microbes and the mind: emerging hallmarks of the gut microbiota-brain axis. Cell Microbiol. 2016;18(5):632–44. Epub 2016/02/27. doi: 10.1111/cmi.12585 . 26918908

[21] Kelly JR, Clarke G, Cryan JF, Dinan TG. Brain-gut-microbiota axis: challenges for translation in psychiatry. Ann Epidemiol. 2016;26(5):366–72. Epub 2016/03/24. doi: 10.1016/j.annepidem.2016.02.008 . 27005587

[22] Osimo EF, Baxter LJ, Lewis G, Jones PB, Khandaker GM. Prevalence of low-grade inflammation in depression: a systematic review and meta-analysis of CRP levels. Psychol Med. 2019;49(12):1958–70. Epub 2019/07/02. doi: 10.1017/S0033291719001454 ; PubMed Central PMCID: PMC6712955. 31258105 PMC6712955

[23] Simpson CA, Diaz-Arteche C, Eliby D, Schwartz OS, Simmons JG, Cowan CSM. The gut microbiota in anxiety and depression—A systematic review. Clin Psychol Rev. 2021;83:101943. Epub 2020/12/04. doi: 10.1016/j.cpr.2020.101943 . 33271426

[24] Stehle JR, Leng XY, Kitzman DW, Nicklas BJ, Kritchevsky SB, High KP. Lipopolysaccharide-Binding Protein, a Surrogate Marker of Microbial Translocation, Is Associated With Physical Function in Healthy Older Adults. J Gerontol a-Biol. 2012;67(11):1212–8. doi: 10.1093/gerona/gls178 PubMed PMID: WOS:000309921500011. 22960476 PMC3636679

[25] Kiecolt-Glaser JK, Wilson SJ, Bailey ML, Andridge R, Peng J, Jaremka LM, et al. Marital distress, depression, and a leaky gut: Translocation of bacterial endotoxin as a pathway to inflammation. Psychoneuroendocrinology 2018;98:52–60. doi: 10.1016/j.psyneuen.2018.08.007 30098513 PMC6260591

[26] Madison AA, Andridge R, Padin AC, Wilson S, Bailey MT, Alfano CM, et al. Endotoxemia coupled with heightened inflammation predicts future depressive symptoms. Psychoneuroendocrinology. 2020;122(1873–3360 (Electronic)):104864. Epub 2020/11/10. doi: 10.1016/j.psyneuen.2020.104864 ; PubMed Central PMCID: PMC7721058. 33166799 PMC7721058

[27] Baker LJ, O’Brien PMS. Shortcomings in RCOG guidance: Green Top Guideline no. 48-Management of Premenstrual Syndrome. Bjog-Int J Obstet Gy. 2013;120(1471–0528 (Electronic)):566-. PubMed PMID: WOS:000320781601649.

[28] Magni LR, Purgato M, Gastaldon C, Papola D, Furukawa TA, Cipriani A, et al. Fluoxetine versus other types of pharmacotherapy for depression. Cochrane Database Syst Rev. 2013;(7):CD004185. Epub 2013/12/20. doi: 10.1002/14651858.CD004185.pub3 . 24353997 PMC11513554

[29] Marjoribanks J, Brown J, O’Brien PM, Wyatt K. Selective serotonin reuptake inhibitors for premenstrual syndrome. Cochrane Database Syst Rev. 2013;(6):CD001396. Epub 2013/06/08. doi: 10.1002/14651858.CD001396.pub3 ; PubMed Central PMCID: PMC7073417. 23744611 PMC7073417

[30] Management of Premenstrual Syndrome: Green-top Guideline No. 48. BJOG. 2017;124(3):e73–e105. Epub 2016/12/03. doi: 10.1111/1471-0528.14260 . 27900828

[31] Takeda T, Tasaka K, Sakata M, Murata Y. Prevalence of premenstrual syndrome and premenstrual dysphoric disorder in Japanese women. Arch Womens Ment Health. 2006;9(4):209–12. Epub 2006/06/09. doi: 10.1007/s00737-006-0137-9 . 16761114

[32] Takeda T, Yoshimi K, Yamada K. Psychometric Testing of the Premenstrual Symptoms Questionnaire and the Association Between Perceived Injustice and Premenstrual Symptoms: A Cross-Sectional Study Among Japanese High School Students. Int J Womens Health. 2020;12:755–63. Epub 2020/10/17. doi: 10.2147/IJWH.S269392 ; PubMed Central PMCID: PMC7524195. 33061664 PMC7524195

[33] Takahashi S, Tomita J, Nishioka K, Hisada T, Nishijima M. Development of a prokaryotic universal primer for simultaneous analysis of Bacteria and Archaea using next-generation sequencing. PLoS One. 2014;9(8):e105592. Epub 2014/08/22. doi: 10.1371/journal.pone.0105592 ; PubMed Central PMCID: PMC4140814. 25144201 PMC4140814

[34] Hisada T, Endoh K, Kuriki K. Inter- and intra-individual variations in seasonal and daily stabilities of the human gut microbiota in Japanese. Arch Microbiol. 2015;197(7):919–34. Epub 2015/06/13. doi: 10.1007/s00203-015-1125-0 ; PubMed Central PMCID: PMC4536265. 26068535 PMC4536265

[35] HG. GA. FASTQ/A short-reads preprocessing tools. 2010.

[36] Sung JY, Hwang Y, Shin MH, Park MS, Lee SH, Yong D, et al. Utility of Conventional Culture and MALDI-TOF MS for Identification of Microbial Communities in Bronchoalveolar Lavage Fluid in Comparison with the GS Junior Next Generation Sequencing System. Ann Lab Med. 2018;38(2):110–8. Epub 2017/12/08. doi: 10.3343/alm.2018.38.2.110 ; PubMed Central PMCID: PMC5736669. 29214754 PMC5736669

[37] Caporaso JG, Kuczynski J, Stombaugh J, Bittinger K, Bushman FD, Costello EK, et al. QIIME allows analysis of high-throughput community sequencing data. Nat Methods. 2010;7(5):335–6. Epub 2010/04/13. doi: 10.1038/nmeth.f.303 ; PubMed Central PMCID: PMC3156573. 20383131 PMC3156573

[38] Edgar RC, Haas BJ, Clemente JC, Quince C, Knight R. UCHIME improves sensitivity and speed of chimera detection. Bioinformatics. 2011;27(16):2194–200. Epub 2011/06/28. doi: 10.1093/bioinformatics/btr381 ; PubMed Central PMCID: PMC3150044. 21700674 PMC3150044

[39] Kasai C, Sugimoto K, Moritani I, Tanaka J, Oya Y, Inoue H, et al. Comparison of the gut microbiota composition between obese and non-obese individuals in a Japanese population, as analyzed by terminal restriction fragment length polymorphism and next-generation sequencing. BMC Gastroenterol. 2015;15:100. Epub 2015/08/12. doi: 10.1186/s12876-015-0330-2 ; PubMed Central PMCID: PMC4531509. 26261039 PMC4531509

[40] Bolyen E, Rideout JR, Dillon MR, Bokulich NA, Abnet CC, Al-Ghalith GA, et al. Reproducible, interactive, scalable and extensible microbiome data science using QIIME 2. Nat Biotechnol. 2019;37(8):852–7. Epub 2019/07/26. doi: 10.1038/s41587-019-0209-9 ; PubMed Central PMCID: PMC7015180. 31341288 PMC7015180

[41] Callahan BJ, McMurdie PJ, Rosen MJ, Han AW, Johnson AJ, Holmes SP. DADA2: High-resolution sample inference from Illumina amplicon data. Nat Methods. 2016;13(7):581–3. Epub 2016/05/24. doi: 10.1038/nmeth.3869 ; PubMed Central PMCID: PMC4927377. 27214047 PMC4927377

[42] DeSantis TZ, Hugenholtz P, Larsen N, Rojas M, Brodie EL, Keller K, et al. Greengenes, a chimera-checked 16S rRNA gene database and workbench compatible with ARB. Appl Environ Microbiol. 2006;72(7):5069–72. Epub 2006/07/06. doi: 10.1128/AEM.03006-05 ; PubMed Central PMCID: PMC1489311. 16820507 PMC1489311

[43] Chao A, Chiu C-H. Nonparametric Estimation and Comparison of Species Richness. eLS2016. p. 1–11.

[44] Shannon CE. A mathematical theory of communication. The Bell System Technical Journal. 1948;27(3):379–423.

[45] Simpson EH. Measurement of diversity. Nature. 1949;163.

[46] Segata N, Izard J, Waldron L, Gevers D, Miropolsky L, Garrett WS, et al. Metagenomic biomarker discovery and explanation. Genome Biol. 2011;12(6):R60. Epub 2011/06/28. doi: 10.1186/gb-2011-12-6-r60 ; PubMed Central PMCID: PMC3218848. 21702898 PMC3218848

[47] Cohen J. A power primer. Psychol Bull. 1992;112(1):155–9. Epub 1992/07/01. doi: 10.1037//0033-2909.112.1.155 . 19565683

[48] Ozato N, Saito S, Yamaguchi T, Katashima M, Tokuda I, Sawada K, et al. Blautia genus associated with visceral fat accumulation in adults 20–76 years of age. NPJ Biofilms Microbiomes. 2019;5(1):28. Epub 2019/10/12. doi: 10.1038/s41522-019-0101-x ; PubMed Central PMCID: PMC6778088. 31602309 PMC6778088

[49] Simpson CA, Mu A, Haslam N, Schwartz OS, Simmons JG. Feeling down? A systematic review of the gut microbiota in anxiety/depression and irritable bowel syndrome. J Affect Disord. 2020;266:429–46. Epub 2020/02/15. doi: 10.1016/j.jad.2020.01.124 . 32056910

[50] Le Chatelier E, Nielsen T, Qin J, Prifti E, Hildebrand F, Falony G, et al. Richness of human gut microbiome correlates with metabolic markers. Nature. 2013;500(7464):541–6. Epub 2013/08/30. doi: 10.1038/nature12506 . 23985870

[51] Ley RE, Turnbaugh PJ, Klein S, Gordon JI. Microbial ecology: human gut microbes associated with obesity. Nature. 2006;444(7122):1022–3. Epub 2006/12/22. doi: 10.1038/4441022a . 17183309

[52] Naseribafrouei A, Hestad K, Avershina E, Sekelja M, Linlokken A, Wilson R, et al. Correlation between the human fecal microbiota and depression. Neurogastroent Motil. 2014;26(8):1155–62. doi: 10.1111/nmo.12378 PubMed PMID: WOS:000339669500011. 24888394

[53] Masho SW, Adera T, South-Paul J. Obesity as a risk factor for premenstrual syndrome. J Psychosom Obst Gyn. 2005;26(1):33–9. doi: 10.1080/01443610400023049 PubMed PMID: WOS:000228743900007. 15962720

[54] Dreimuller N, Lieb K, Tadic A, Engelmann J, Wollschlager D, Wagner S. Body mass index (BMI) in major depressive disorder and its effects on depressive symptomatology and antidepressant response. J Affect Disorders. 2019;256(1573–2517 (Electronic)):524–31. doi: 10.1016/j.jad.2019.06.067 PubMed PMID: WOS:000491354400066. 31280076

[55] Hashizume K, Tsukahara T, Yamada K, Koyama H, Ushida K. Megasphaera elsdenii JCM1772T normalizes hyperlactate production in the large intestine of fructooligosaccharide-fed rats by stimulating butyrate production. The Journal of nutrition. 2003;133(10):3187–90. Epub 2003/10/02. doi: 10.1093/jn/133.10.3187 . 14519808

[56] Schroeder FA, Lin CL, Crusio WE, Akbarian S. Antidepressant-like effects of the histone deacetylase inhibitor, sodium butyrate, in the mouse. Biol Psychiatry. 2007;62(1):55–64. Epub 2006/09/02. doi: 10.1016/j.biopsych.2006.06.036 . 16945350

[57] Zhou Y, Chen C, Yu H, Yang Z. Fecal Microbiota Changes in Patients With Postpartum Depressive Disorder. Front Cell Infect Microbiol. 2020;10(2235–2988 (Electronic)):567268. Epub 2020/11/03. doi: 10.3389/fcimb.2020.567268 ; PubMed Central PMCID: PMC7550660. 33134190 PMC7550660

[58] Kikuchi S, Kobayashi N, Watanabe Z, Ono C, Takeda T, Nishigori H, et al. The delivery of a placenta/fetus with high gonadal steroid production contributes to postpartum depressive symptoms. Depress Anxiety. 2021;38(4):422–30. Epub 2021/01/05. doi: 10.1002/da.23134 . 33393686

[59] Buttner MM, Mott SL, Pearlstein T, Stuart S, Zlotnick C, O’Hara MW. Examination of premenstrual symptoms as a risk factor for depression in postpartum women. Arch Womens Ment Health. 2013;16(3):219–25. Epub 2013/01/09. doi: 10.1007/s00737-012-0323-x ; PubMed Central PMCID: PMC3663927. 23296333 PMC3663927

[60] Cekanaviciute E, Yoo BB, Runia TF, Debelius JW, Singh S, Nelson CA, et al. Gut bacteria from multiple sclerosis patients modulate human T cells and exacerbate symptoms in mouse models. Proc Natl Acad Sci U S A. 2017;114(40):10713–8. Epub 2017/09/13. doi: 10.1073/pnas.1711235114 ; PubMed Central PMCID: PMC5635915. 28893978 PMC5635915

[61] Olson CA, Vuong HE, Yano JM, Liang QY, Nusbaum DJ, Hsiao EY. The Gut Microbiota Mediates the Anti-Seizure Effects of the Ketogenic Diet. Cell. 2018;173(7):1728–41 e13. Epub 2018/05/29. doi: 10.1016/j.cell.2018.04.027 ; PubMed Central PMCID: PMC6003870. 29804833 PMC6003870

[62] Wang K, Liao M, Zhou N, Bao L, Ma K, Zheng Z, et al. Parabacteroides distasonis Alleviates Obesity and Metabolic Dysfunctions via Production of Succinate and Secondary Bile Acids. Cell Rep. 2019;26(1):222–35 e5. Epub 2019/01/04. doi: 10.1016/j.celrep.2018.12.028 . 30605678

[63] Koh GY, Kane A, Lee K, Xu Q, Wu X, Roper J, et al. Parabacteroides distasonis attenuates toll-like receptor 4 signaling and Akt activation and blocks colon tumor formation in high-fat diet-fed azoxymethane-treated mice. Int J Cancer. 2018;143(7):1797–805. Epub 2018/04/27. doi: 10.1002/ijc.31559 . 29696632

[64] Strandwitz P, Kim KH, Terekhova D, Liu JK, Sharma A, Levering J, et al. GABA-modulating bacteria of the human gut microbiota. Nat Microbiol. 2019;4(3):396–403. Epub 2018/12/12. doi: 10.1038/s41564-018-0307-3 ; PubMed Central PMCID: PMC6384127. 30531975 PMC6384127

[65] Craner JR, Sigmon ST, McGillicuddy ML. Does a disconnect occur between research and practice for premenstrual dysphoric disorder (PMDD) diagnostic procedures? Women Health. 2014;54(3):232–44. Epub 2014/02/12. doi: 10.1080/03630242.2014.883658 . 24512469

[66] Takeda T, Kai S, Yoshimi K. Psychometric Testing of the Japanese Version of the Daily Record of Severity of Problems Among Japanese Women. International Journal of Womens Health. 2021;13(1179–1411 (Print)):361–7. doi: 10.2147/IJWH.S301260 PubMed PMID: WOS:000637042400001. 33833588 PMC8020050

