# Supplementary material for: Characteristics of the gut microbiota in women with premenstrual symptoms: A cross-sectional study
Source: PLoS One. 2022 May 27;17(5):e0268466. doi: 10.1371/journal.pone.0268466 (PMC9140228; doi:10.1371/journal.pone.0268466)
Supplement: Attachment — Submitted filename: Response to Reviewrs.docx [file pone.0268466.s006.docx]

Professor Emily Chenette

Editor-in-Chief

PLOS ONE

March 24, 2022

Dear Professor Chenette,

Thank you for reconsidering our manuscript entitled “Characteristics of the gut microbiota in women with premenstrual symptoms: A cross-sectional study” as an Original Research article for publication in PLOS ONE. We have revised our manuscript in response to the many helpful comments of the reviewers, as shown below.

**Additional Editor Comments:**

*The primary remaining issue is data availability. Please see the policy of PLOS ONE: https://journals.plos.org/plosone/s/data-availability.*

*“If there are ethical or legal restrictions on sharing a sensitive data set, authors should provide the following information within their Data Availability Statement upon submission:*

*• Explain the restrictions in detail (e.g., data contain potentially identifying or sensitive patient information)*

*• Provide contact information for a data access committee, ethics committee, or other institutional body to which data requests may be sent”*

*“Please note it is not acceptable for an author to be the sole named individual responsible for ensuring data access.”*

*If your university has imposed restrictions on sharing of this data, you must provide the contact information for a data access committee (or equivalent institutional*

*committee) that is authorized to review data requests and share dataupon approval. One of the authors cannot be named as being responsible for data requests.*

Re: According to this comment, we have added the relevant information as supporting data in files S1 to S4.

**Reviewer: 1**

*All the comments and concerns have been satisfactorily addressed except for the data availability issue.*

Re: Thank you for appreciating our efforts. We have added the requested information as supporting data in files S1 to S4.

**Reviewer: 2**

*1. Data Sharing (Major concern): The authors have not followed the PLOS Data policy which requires authors to make all data underlying the findings described in their manuscript fully available without restriction. The authors cited “potentially sensitive patient information” as the rationale for not making the data available. It is unclear how deidentified patients’ data contain any potentially sensitive patient information. It is also questionable whether a data access request from a fellow researcher will be honored in the future.*

Re: Initially, we thought that the data could not be made public because they were clinical data. However, as you point out, the data are completely anonymized and thus not individuals are not identifiable. Therefore, we have made the data available by including it as supporting information in files S1 to S4.

Introduction

*2. The added hypothesis was “dysbiosis of the microbiota is associated with pathogenesis of PMDs.” Please define “dysbiosis.” Please stay away from causal language as this small cross-sectional study could not establish causation.*

Re: As stated in the limitations, we understand that it is not possible to state causality based on the results of this study. We have revised the manuscript to avoid any causal language. The purpose of our study was to explore the relationship between the gut microbiota and PMDs in an exploratory manner. To make this clear, we have revised the text on page 5, lines 87–89.

*3. The authors used the term “low grade inflammation.” What does it mean? What are the characteristics/markers of low grade inflammation?*

Re: In reference 22, low grade inflammation is defined as a CRP level of greater than 3 mg/L. We have added this information on page 5, lines 75–76.

Methods:

*4. I am puzzled by the fact that 7 symptom-free women reported “symptom interference with social life”. Please*

*explain.*

Re: We apologize for the lack of clarity in our explanation. As we describe in the Introduction on page 4, line 56, the prevalence of premenstrual symptoms is high (80%–90%), so completely symptom-free women are rare. The inclusion criteria for the N group included no symptoms listed in the PSQ showing a moderate or higher level, as described on page 6, lines 112–113, so this means that the N group includes those with mild premenstrual symptoms. These women are not completely free of premenstrual symptoms. The seven patients excluded from the N group had multiple mild premenstrual symptoms and mild interference of social life due to these symptoms as assessed by the PSQ. To clarify, we have revised the text on page 7, lines 124–128. In addition, we changed the wording of the description of the N group in Fig. 1 from “Not reporting premenstrual symptoms” to “Not reporting serious premenstrual symptoms”. For consistency, the text on page 2, line 26, 28 and on page 6, line 105, was also changed accordingly.

*5. Some details on sample collection are missing. What is the length of time from collection to receipt by the lab? Participant compliance can be an issue when you ask participants to store stool samples in 4 Celsius degrees (in their fridge??). There can be issues related to sample integrity. Please comment how you approached participant compliance or acknowledge this as a limitation.*

Re: In this study, special sampling tubes were used for DNA collection from stool specimens. Within these tubes was a storage solution containing guanidine, which is guaranteed to be stable for one month at room temperature or 4 °C; therefore, subjects did not need to store their specimens in a refrigerator. We have added this information on page 9, lines 177–182.

*6. Please describe the rationale to assess bacteria translocation indirectly rather than directly.*

Re: Analyzing the DNA of intestinal bacteria in blood is more labor-intensive and costly than indirect marker measurements. Given the exploratory nature of this study, we chose to examine bacterial translocation via an indirect method. We have added this explanation on page 18, lines 342–344.

*7. Those who took drospirenone-containing oral contraceptives were excluded from the study. What about other types of hormonal contraceptives? Did you collect the data on those and consider them when interpreting the findings?*

Re: Drospirenone-containing OCs were excluded because, unlike other conventional OCs, they have a proven therapeutic effect on PMDs. The percentage of Japanese women who use OCs as a contraceptive method is low (0.9%, as reported by the WHO in 2015), and as a result, none of the subjects in this study were OC users. We have added additional information to explain this on page 6, lines 110–111 and 117.

Results

*8. It is unclear why LEfSE plots could not be used.*

Re: In the first revision, we seem to have misunderstood the reviewer's comments on “LEfSE plots”. Assuming that "LEfSE plots" refers to cladograms, we have added these data as a new Fig 4A. We also changed the figure legend for Fig 4 accordingly on page 15, lines 300–302 and page 16, lines 303–306.

.

*9. Can you report the effect size between groups for gut microbiome data?*

Re: We analyzed the effect sizes and have added these data to Fig 3.

Discussion

*10. Please explain the negative findings regarding alpha diversity.*

Re: Alpha diversity was already described in the original version of the study in the case of MDD and inflammatory bowel disease, but a comparison with the PMD results obtained here was missing. Therefore, an explanation of alpha diversity relating to PMDs and MDD was added on page 18, line 354.

Overall

*11. The manuscript can benefit from proof-reading by an English*

*editor.*

Re: Our revised manuscript has been reviewed by a native English-speaking expert editor.

We hope our revised manuscript is now acceptable for publication in *PLOS ONE.* We look forward to hearing from you at your earliest convenience.

Yours sincerely,

Takashi Takeda, MD, PhD
