# Supplementary material for: Characteristics of the gut microbiota in women with premenstrual symptoms: A cross-sectional study
Source: PLoS One. 2022 May 27;17(5):e0268466. doi: 10.1371/journal.pone.0268466 (PMC9140228; doi:10.1371/journal.pone.0268466)
Supplement: Attachment — Submitted filename: Response to Reviewrs R3.docx [file pone.0268466.s007.docx]

Professor Emily Chenette

Editor-in-Chief

PLOS ONE

April 28, 2022

Dear Professor Chenette,

Thank you for reconsidering our manuscript entitled “Characteristics of the gut microbiota in women with premenstrual symptoms: A cross-sectional study” as an Original Research article for publication in PLOS ONE. We have revised our manuscript in response to the helpful comments of the editor, as shown below.

**Additional Editor Comments:**

*The authors have adequately addressed the comments on the prior revision of their manuscript, with the exception that the data made available consists of derived datasets (genus and phylum level count table) rather than raw sequence data as is standard in the field (i.e. fastq files). Could the authors deposit the raw sequence data in a public repository (e.g. NCBI SRA) or provide a reason why the raw sequence data are no longer available?*

Re: Following this comment, we registered the raw sequence data to the public repository (DRA) and added the accession numbers on page 23, line 442–445.

In this revision, we reviewed the description of the method section and found some mistakes. Therefore, we have revised it as described on page 10, line 202–204.

We hope our revised manuscript is now acceptable for publication in *PLOS ONE.* We look forward to hearing from you at your earliest convenience.

Yours sincerely,

Takashi Takeda, MD, PhD
